# Supplementary material for: Persistent eczema leads to both impaired growth and food allergy: JECS birth cohort
Source: PLoS One. 2021 Dec 1;16(12):e0260447. doi: 10.1371/journal.pone.0260447 (PMC8635351; doi:10.1371/journal.pone.0260447)
Supplement: S1 Fig — (DOCX) [file pone.0260447.s001.docx]

Fetal records

n = 104,062

Live birth

n = 100,304

Term birth (37weeks and more)

n = 94,427

Singleton baby

n = 93,507

Without neonatal complications

n = 86,200

Healthy child without chronic disease at 2 years old

n = 66,069

Healthy child without chronic disease at 3 years old

n = 59,847

Miscarriage, n = 1,245

Stillbirth, n = 382

No response, n = 2,122

Preterm baby (<37 weeks), n = 5,584

No response, n = 293

Multiple baby, n = 920

Neonatal complication, n = 5,455

No response, n = 1,852

Chronic disease at 2 years old, n = 8,243

No response, n = 11,888

Chronic disease at 3 years old, n = 1,610

No response, n = 4,612

S1 Fig
